# P-919. Epidemiology of Cerebral Sinovenous Thrombosis in Children with Acute Bacterial Intracranial Infection: a Single-Institution Retrospective Study

**DOI:** 10.1093/ofid/ofae631.1110

**Published:** 2025-01-29

**Authors:** Nehali Mehta, Sanjeev K Swami, Mark Halverson, Leah Loerinc, Claudia Gambrah-Lyles, Jennifer McGuire

**Affiliations:** Children's Hospital of Philadelphia, Philadelphia, Pennsylvania; Children's Hospital of Philadelphia, Philadelphia, Pennsylvania; Children's Hospital of Philadelphia, Philadelphia, Pennsylvania; Children's Hospital of Philadelphia, Philadelphia, Pennsylvania; Children's Hospital of Philadelphia, Philadelphia, Pennsylvania; Children's Hospital of Philadelphia, Philadelphia, Pennsylvania

## Abstract

**Background:**

Cerebral sinovenous thrombosis (CSVT) is a known complication of acute bacterial intracranial infection. The objective of this study is to determine the annual proportion of, risk factors for, and outcomes in children with CSVT secondary to acute bacterial intracranial infection.

**Figure 1:**
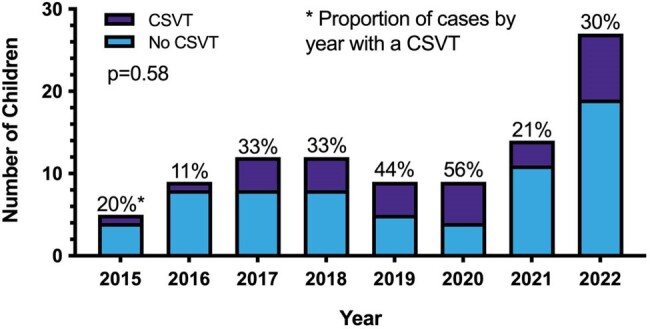
Annual proportion of CSVT in children with acute bacterial intracranial infection between Jan 1, 2015 and December 31, 2022

**Methods:**

Retrospective single-center cohort study of children age 1-18 years hospitalized at a tertiary care children’s hospital for acute bacterial intracranial infection between 01/01/2015 and 02/01/2023. Cases were identified by discharge ICD codes for “meningitis,” “meningoencephalitis,” “subdural empyema,” “epidural abscess,” and “brain abscess.” Medical charts were manually screened to ascertain the diagnosis and to abstract relevant clinical data. Multivariate models were built to examine risk factors for CSVT.

**Figure:**
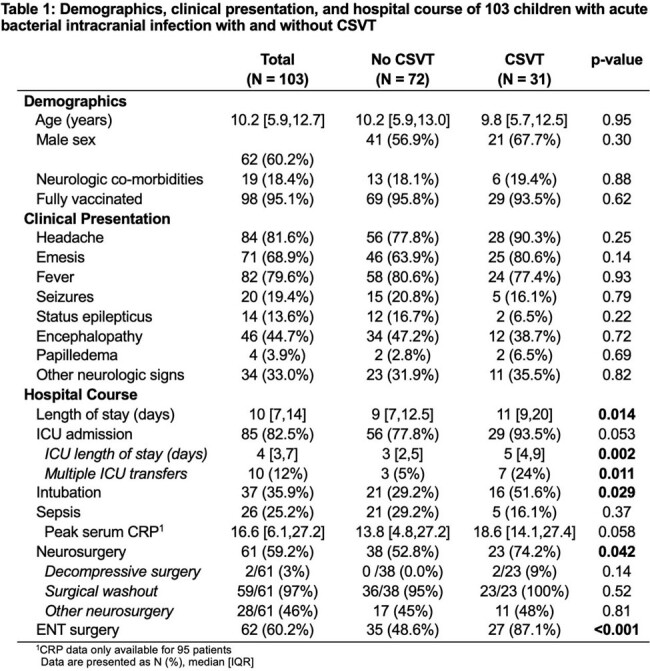

**Results:**

One hundred and three patients were included. Median age was 10.2 [5.9,12.7] years. 31/103 (30%) had CSVT. Annual proportion of CSVT did not vary; however, the total number of cases of intracranial infection rose during the study period. Patients with CSVT were no different from those without CSVT by demographics, vaccination status, neurologic signs or symptoms at presentation, or causative organisms. Non-pneumococcal *streptococcus* species were the most common causative organisms, found in 37/103 (36%) children. Need for neurosurgical intervention (aOR=11.6, p=0.027), need for ENT intervention (aOR=6.1, p=0.013), temporal location of infection (aOR=7.9, p=0.004), and concurrent mastoiditis (aOR=29.5, p=0.004) were associated with CSVT. The sigmoid sinus (55%, 17/31), transverse sinus (42%, 13/31), jugular vein (45%, 14/31), and superior sagittal sinus (45%, 14/31) were the most common locations for CSVT. Two patients (6%) had venous infarction; one (3%) had venous hemorrhage. A higher proportion of children with CSVT had behavioral concerns (p=0.020) and learning concerns (p=0.025) than children without CSVT.

**Figure:**
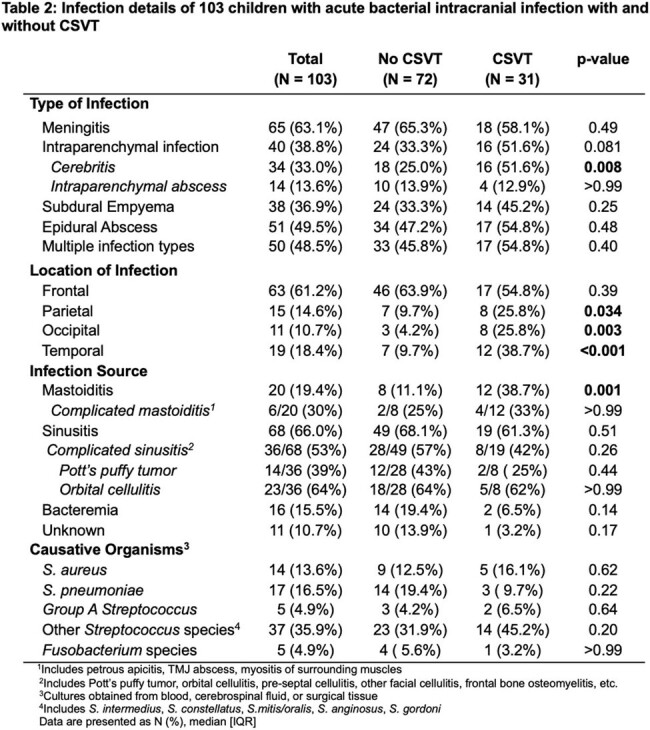

**Conclusion:**

CSVT is common in acute bacterial intracranial infection. Consider empiric surveillance in children with acute bacterial intracranial infection with mastoiditis, temporal location of infection, and/or those who require surgical intervention. Outcomes in this population should be further explored.

**Figure:**
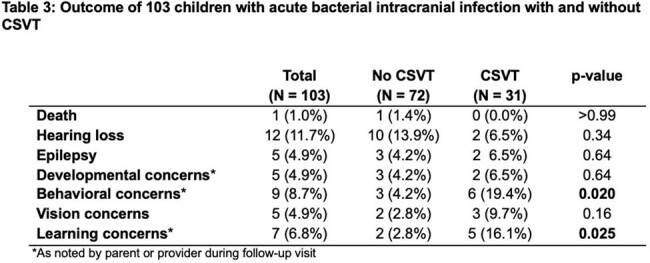

**Disclosures:**

**All Authors**: No reported disclosures

